# Massive Open Online Courses for Health Worker Education in Low- and Middle-Income Countries: A Scoping Review

**DOI:** 10.3389/fpubh.2022.891987

**Published:** 2022-07-12

**Authors:** Jessica Nieder, Patricia Nayna Schwerdtle, Rainer Sauerborn, Sandra Barteit

**Affiliations:** ^1^Heidelberg Institute of Global Health (HIGH), Faculty of Medicine and University Hospital, Heidelberg University, Heidelberg, Germany; ^2^Nursing and Midwifery, Faculty of Medicine, Nursing and Health Science, Monash University, Clayton, VIC, Australia

**Keywords:** Massive Open Online Course, health care workers, education, low- and middle-income countries, health professions education, medical education

## Abstract

**Background:**

Massive Open Online Courses (MOOCs) have the potential to improve access to quality education for health care workers (HCWs) globally. Although studies have reported on the use of MOOCs in low- and middle-income countries (LMICs), our understanding of the scope of their utilization or access barriers and facilitators for this cohort is limited. We conducted a scoping review to map published peer-reviewed literature on MOOCs for HCW education in LMICs. We systematically searched four academic databases (Scopus, Web of Science, PubMed, ERIC) and Google Scholar, and undertook a two-stage screening process. The analysis included studies that reported on MOOCs relevant to HCWs' education accessed by HCWs based in LMICs.

**Results:**

The search identified 1,317 studies with 39 studies included in the analysis, representing 40 MOOCs accessed in over 90 LMICs. We found that MOOCs covered a wide range of HCWs' including nurses, midwives, physicians, dentists, psychologists, and other workers from the broader health care sector, mainly at a post-graduate level. Dominant topics covered by the MOOCs included infectious diseases and epidemic response, treatment and prevention of non-communicable diseases, communication techniques and patient interaction, as well as research practice. Time contribution and internet connection were recognized barriers to MOOC completion, whilst deadlines, email reminders, graphical design of the MOOC, and blended learning modes facilitated uptake and completion. MOOCs were predominantly taught in English (20%), French (12.5%), Spanish (7.5%) and Portuguese (7.5%). Overall, evaluation outcomes were positive and focused on completion rate, learner gain, and student satisfaction.

**Conclusion:**

We conclude that MOOCs can be an adequate tool to support HCWs' education in LMICs and may be particularly suited for supporting knowledge and understanding. Heterogeneous reporting of MOOC characteristics and lack of cohort-specific reporting limits our ability to evaluate MOOCs at a broader scale; we make suggestions on how standardized reporting may offset this problem. Further research should focus on the impact of learning through MOOCs, as well as on the work of HCWs and the apparent lack of courses covering the key causes of diseases in LMICs. This will result in increased understanding of the extent to which MOOCs can be utilized in this context.

## Introduction

The ongoing COVID-19 pandemic has underlined the importance of well-trained health care workers (HCWs) in a functioning health care system (1, 2). However, there is a growing global concern regarding the availability of HCWs, as the World Health Organization (WHO) predicts a shortfall of 18 million HCWs by 2030, most of whom will be missing in low-income and middle-income countries (LMICs), especially in rural areas (1, 3). In particular, these countries are faced with a triple burden of disease through the maintained burden of infectious diseases, as well as non-communicable diseases and health impacts through climate change which are likely to negatively affect weak health systems even more (4). Inadequate resources and infrastructure, such as training facilities, educators, and financing for continued professional development (CPD), all contribute to the shortage of HCW in LMICs (5–7). Scaling up and increasing access to quality HCW education is critical to increasing the number of qualified HCWs needed to provide sufficient health care in LMICs.

Over the past two decades, Massive Open Online Courses (MOOCs) have emerged as a mode of electronic learning (e-learning) (8, 9), fostered by steadily increasing internet access (10). MOOCs are courses that can be taken by a theoretically unlimited number of participants (massive), without cost or formal admission (open), that are accessible *via* the internet (online) whilst following a structured learning plan (course) (11). Due to the capacity of MOOCs to simultaneously reach a large number of learners regardless of geographical location, their open-access nature, and their attraction to students from diverse educational backgrounds, MOOCs may provide a means to increase access to quality education for HCWs (12–14). Since the introduction of the MOOC concept in 2008 (15), interest has increased over the years, reflected in rising course offerings and a continuous high volume of learners (16). MOOCs are now offered by over 950 institutions including many prestigious universities and accessed by over 180 million learners from all parts of the world (16). Overall, MOOCs cover a wide range of topics and in 2020, 7.7% of all available MOOCs covered the field of health and medicine (16).

Several case studies have reported on the successful use of MOOCs for HCWs' education specifically in LMICs. For example, the World Health Organization (WHO) uses the openWHO platform to provide MOOCs that aim to quickly spread knowledge about responses to emerging diseases such as the Plague, Diphtheria, Ebola (17), and COVID-19 (18) to a large number of people. Evaluations on the reach of these courses show high uptake within outbreak countries (17, 18). Similarly, the Latin American Nephrology community has developed a MOOC on kidney immunopathology, targeting all HCWs involved in kidney transplantation in Latin America and concluded that MOOCs are a potential tool for health workers' professional development and reduce heterogeneity in their access to training resources (19). Following a review on the global use of MOOCs for nurses and other health care workers, Longhini et al. (20) concluded that massive open online education may be suitable when face-to-face education is not possible or to reach a broad audience in a short period of time, supporting the notion that MOOCs may be used in HCWs' education to support existing structures by making education more readily available (21). In contrast, Rowe et al. (22) concluded that there is insufficient evidence to advocate their usage for educating medical students. In addition to these findings, we found limitations in the current literature on differences in MOOC use across high- and low- and middle-income countries, as no reviews have differentiated between the use of MOOCs in high and low-and middle income countries. Facilitators and barriers of MOOCs and their capacity to address the educational needs of HCWs in LMIC settings remain unclear. Therefore, to map the state of evidence regarding how MOOCs are used for HCWs' education in these countries and to address the question of the extent to which MOOCs can support the education of HCWs there, we limited the scope of this review to MOOCs accessed by HCWs in LMICs to understand:

- What are the characteristics of MOOCs for health care workers in low- and middle-income countries?
- In which low- and middle-income countries were MOOCs for health care workers accessed by learners?
- What are the content topics covered by MOOCs?
- What are facilitators and barriers to educating health care workers with MOOCs in low- and middle-income countries?
- How MOOCs are evaluated?

## Methods

Due to the broad scope of the research questions and the aim to include all types of studies, a scoping literature review seemed most adequate (23). This scoping review was underpinned by the methodological framework developed by Arksey and O'Malley (19) and advanced by Levac et al. (24). Accordingly, the review underwent five stages, iteratively: (1) Identify the research question, (2) Identify relevant studies, (3) Select relevant studies, (4) Chart the data, (5) Collect, summarize, and report results (19). The review is reported in line with the Preferred Reporting Items for Systematic Reviews and Meta-Analysis – Scoping Review (PRISMA-ScR) (25) framework.

### Search Strategy

We systematically searched four academic databases, namely Web of Science, Scopus, PubMed, and Eric. Web of Science and Scopus were chosen for their broad coverage, whilst PubMed and Eric were chosen for their subject-specific focus on health, medicine, and education, respectively. To identify gray literature and any articles not included in the academic databases, we also searched Google Scholar and screened the first 1,000 search results (26).

We developed the search strategy, with the assistance of a university-based librarian, from the two core concepts; “*MOOCs”* and “*health professional education”* without specifying level of income or country to ensure the search was as inclusive as possible. We identified synonyms and Medical Subject heading (MeSH) terms and further keywords based on test searches and adapted the final search string to fit the syntax requirements of each database (see Table 1 for the search string conducted in Scopus as an example and Supplementary Appendix 1 for detailed search strings and search queries for the respective databases).

**Table 1:** Search string for Scopus.

**Database**	**Search string**
Scopus	TITLE-ABS-KEY (mooc* OR “Massive Open Online Course”) AND TITLE-ABS-KEY (health* OR “public health” OR healthcare OR “health professional” OR “health care worker” OR “health personnel” OR “allied health personnel” OR “human resources for health” OR “health care provider” OR “health occupation” OR “allied health occupation” OR nurse* OR doctor* OR midwife OR dietician* OR “medical education” OR “health education” OR “medical student” OR “Allied health occupation” OR “community health worker”)

### Study Selection and Eligibility

Relevant studies were selected using a two-step procedure: (1) title and abstract screening, (2) full-text screening, using Covidence software (27). To reduce bias, articles were screened individually by two reviewers (JN, PNS), full-text screening was conducted only by the first author, as is common practice for scoping reviews (28). Any conflicts were resolved by a third reviewer (SB). If additional information was required for the study selection, we contacted the first author *via* email.

### Inclusion and Exclusion Criteria

The screening process was guided by inclusion and exclusion criteria which we developed using the adapted population-exposure-outcome (PEO) framework (see Table 2). Studies were included if they reported on a MOOC relevant to HCWs' education. Participants included HCWs (but may have included other participants, i.e., if the MOOC was relevant to other groups), and at least one participant was from an LMIC as defined by the World Bank (29, 30). For the purpose of this review, we define HCWs to include all those whose primary professional aim is to maintain or improve the health of others (31, 32). We interpreted this to include those who provide direct care for patients including nurses, midwives, physiotherapists, psychologists, and physicians. As community health workers play an important role in providing health care in LMICs (33), we did not exclude these even though they may lack formal education. Family carers, social workers, life science professionals, and administrative staff were excluded. As we aim to look at HCWs' education we extended the definition to include those in specialty training. Education for HCWs refers to any education relevant to HCWs, pursued at any level of their career including undergraduate, postgraduate, and CPD. Relevance for HCWs was determined either when the target audience was defined in the article and aligned with our inclusion criteria or if it covered content (knowledge, attitudes, or skills) deemed by the authors as relevant to HCWs' education. We included topics such as patient communication, skill improvement, and disease perception, as well as research for implementation of clinical trials or updating professional skills and excluded courses on management, healthful behavior and lifestyle, mental health management for HCWs, and research not directly applicable to patient care. Finally, LMICs included all countries defined as low-income- (LICs), lower-middle-income-(LMI), or upper-middle-income countries by the World Bank in January 2021 (29, 30, 34). As the term “MOOC” was coined in 2008 (15), and the full search was conducted on 9th December 2020, studies published before 1st January 2008 and after 9th December 2020 were excluded. We did not include pre-prints (i.e., articles that had been accepted for publication but were not yet published). Our search results included two thesis documents. As we only considered original data, secondary and synthesis research were excluded. No studies were excluded based on their study design and only full-text articles were included (see Table 2).

**Table 2:** Inclusion and exclusion criteria based on the population-exposure-outcome framework.

	**Inclusion**	**Exclusion**
**Population**	HCWs^*^ Include at least one participant from an LMIC^**^	Non-HCWs Learner location not stated All learners from HICs
**Exposure**	MOOCs focusing on HCWs' education	MOOCs not focusing on HCW education
**Outcome**	Studies reporting use of the MOOC in at least one LMIC	Studies in which the MOOC was only planned not implemented
**Time**	Published after 1st January 2008	Published before 1st January 2008 Published after 9th December 2020
**Study type**	Any primary research	Secondary/synthesis research
	Gray literature included	
	Full text available	Full text not available
**Language**	English	Languages other than English

* *Health care workers included health professionals and health associate professionals as defined by the WHO (35)*.

** *LMICs as defined by the World Bank as of January 2021*.

### Charting the Data

Extraction codes were created mostly a priori and in accordance with the study objectives; however, some changes were made during the extraction process to accommodate additional important findings. We extracted the author, title, and year of publication as well as characteristics of MOOCs, including the country the MOOC was developed in, course length, hosting platform, teaching language, topic, and targeted audience; the number of participants from LMICs; and any evaluative factors, facilitators, and barriers (see Table 3 for a full list of extraction criteria and their description and Supplementary Appendix 2 for summary extraction sheet).

**Table 3:** Full list of extraction criteria and their description.

**Extraction code**	**Description**
Author, title, year of publication	
MOOC development	Country, MOOC was developed in
Learners' country of residence	
Number of participants from LMICs	
Topic, level, & depth	Health-related topics covered in MOOC learning objectives
Target audience	Target audience
Language	Course language (audio and subtitles)
Length	Course length and time investment by learners
Platform	Web-based platform hosting the MOOC platform
Participation cost	Access cost/barriers
Method of delivery	MOOC
Credits given	Course credits
Evaluation	Factors used to evaluate the course in any way. Authors conclusion
Barriers and facilitators	Barriers or challenges identified by authors Factors facilitating uptake
Learner feedback	Feedback given by learners

## Results

**Figure 1:**
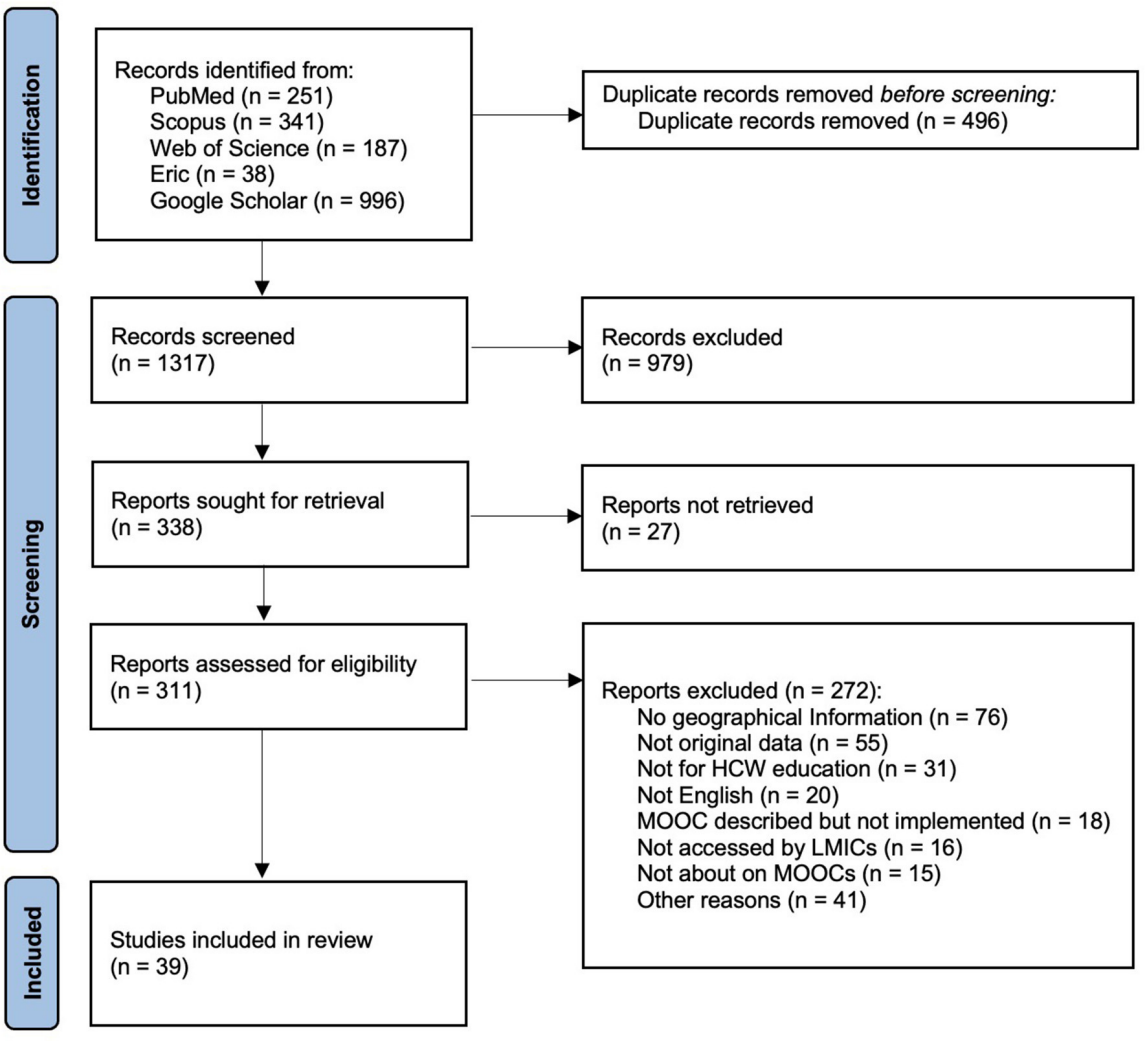
PRISMA flow diagram.

The systematic search of the databases returned 1,813 articles. Following title and abstract screening and removal of duplicates, 332 studies were included in the full-text review; 39 met the inclusion criteria and were included in the scoping review analysis. The main reasons for exclusion were learner location not reported or not clearly stated, MOOC not targeted at HCWs, and publications that did not include original data (Figure 1). The 39 included studies reported on 40 different MOOCs, a summary of the extracted data is available in Supplementary Appendix 2.

### Characteristics of Included MOOCs

Most MOOCs were developed by institutions from either higher income (*n* = 14, 35%) or upper middle income countries (*n* = 9, 22.5%). Four MOOCs (10%) were developed by the WHO, and seven (15.5%) were multi-country efforts with participation from HICs (for details see Table 4).

**Table 4:** MOOC characteristics of included studies.

**Characteristics**	No. of MOOCs (%)^*^	**References**
**MOOC development**		
Multinational (HIC-LIC)	7 (17.5%)	(36–42)
HIC collaboration	3 (7.5%)	(42–44)
WHO	4 (10%)	(17, 18)
UK	4 (10%)	(45–48)
USA	5 (12.5%)	(49–53)
Sweden	3 (7.5%)	(54–56)
Canada	1 (2.5%)	(57)
Brazil	3 (7.5%)	(58–60)
China	3 (7.5%)	(61–63)
Mexico	2 (5%)	(64, 65)
South Africa	1 (2.5%)	(66)
Switzerland	1 (2.5%)	(67)
Not specified	3 (7.5%)	(68, 69)
**MOOC platform**		
edX	6 (15%)	(41, 52, 54–56, 69)
Coursera	5 (12.5%)	(49, 50, 53, 67, 68)
openWHO	4 (10%)	(17, 18)
Future Learn	3 (7.5%)	(47, 48, 66)
iversity	1 (2.5%)	(42)
FUN	1 (2.5%)	(42)
Lcourse163	1 (2.5%)	(61)
Other	6 (15%)	(36, 38, 39, 44, 51, 57, 70, 71)
Not specified	14 (35%)	(37, 40, 43, 45, 46, 58–60, 62–65, 72)
Language^**^		
English	10 (25%)	(18, 39, 42, 43, 45, 48–50, 53, 66, 70, 71)
French	5 (12.5%)	(17, 18, 42, 57)
Spanish	3 (7.5%)	(18, 36, 38)
Portuguese	3 (7.5%)	(18, 36, 38)
Lingala	1 (2.5%)	(17)
Chinese	1 (2.5%)	(18)
Russian	1 (2.5%)	(18)
Arabic	1 (2.5%)	(18)
Hindi	1 (2.5%)	(18)
Turkish	1 (2.5%)	(18)
Persian	1 (2.5%)	(18)
Serbian	1 (2.5%)	(18)
Indian sign	1 (2.5%)	(18)
Not specified	29 (72.5%)	(37, 41, 44, 46, 47, 51, 52, 54–56, 58–65, 67–69, 72)
Subtitles^**^		
English	4 (10%)	(40, 42, 48, 55)
Spanish	3 (7.5%)	(40, 42, 48)
French	2 (5%)	(36, 49)
Chinese-Mandarin	1 (2.5%)	(48)
Russian	1 (2.5%)	(42)
Arabic	1 (2.5%)	(42)
Hindi	1 (2.5%)	(42)
Indonesian	1 (2.5%)	(42)
Portuguese	1 (2.5%)	(42)
Not specified	36 (90%)	
**Access cost**		
Free	26 (65%)	(17, 18, 42, 43, 45, 47, 49, 51, 52, 58, 60, 66, 70)
Access *via* invitation only	1 (2.5%)	(40)
Access cost	1 (2.5%)	(36)
Not specified	12 (30%)	
**Teaching format**		
Blended teaching	5 (12.5%)	(52, 61, 63, 64, 67)
Integration into formal University training	4 (10%)	(59, 61, 63, 71)
**Target audience**		
Without requirements	9 (22.5%)	(37, 42, 46, 50, 56, 57, 72)
Undergraduates	9 (22.5%)	(41, 49, 52, 54, 59–62, 71)
Postgraduates	22 (56%)	(36, 40, 43, 44, 47, 49, 50, 58, 60, 64, 68–70)
**Certificate**		
CPD credits	5 (12.5%)	(39, 51, 52, 58, 70)
Certificate of participation or completion	20 (50%)	(38, 40–44, 48, 50, 52, 53, 55–57, 63–65, 68–70)
No certificate	4 (10%)	(17, 18)
Not specified	10 (25%)	(37, 45–47, 49, 59, 61, 66, 71, 72)
**Publication date**		
2014	2 (5%)	(39, 51)
2015	3 (7.5%)	(38, 49, 71)
2016	4 (10%)	(47, 66, 67, 69)
2017	9 (22.5%)	(36, 45, 50, 55, 56, 59, 63, 68, 70)
2018	7 (17.5%)	(17, 41, 42, 48, 54, 64, 65)
2019	7 (17.5%)	(37, 44, 46, 52, 58, 61, 72)
2020	7 (17.5%)	(18, 40, 43, 53, 57, 60, 62)

* *Based on n = 40 MOOCs*.

** *Some MOOCs were offered in multiple languages*.

The teaching language was reported for 20 MOOCs (20%) and was most often (*n* = 10, 25%) English followed by French (*n* = 6, 15%), Spanish (*n* = 3, 7.5%) and Portuguese (*n* = 3, 7.5%). Three MOOCs (7.5%) used subtitles in languages other than English and one (2.5%) had English subtitles.

EdX and Coursera were the most frequent MOOC platforms, hosting six (15%) and five (12.5%) of the included MOOCs respectively. This was followed by openWHO with four (10%), FutureLearn with three (7.5%), and iversity, Fun MOOC, and icourse163 with one (2.5%) MOOC each. Six MOOCs (15%) were hosted on other online platforms. For the remaining 14 MOOCs (35%), the specific online platform was not reported.

Five (12.5%) MOOCs were embedded in blended learning approaches, providing MOOC participants with the option of attending face-to-face sessions. Two (5%) were specifically developed to be part of a university degree, while another MOOC (2.5%) was suggested within a university degree but was not developed specifically for this context.

By definition, all MOOCs should be open access, however, only 26 (65%) studies specifically reported being free of cost for participants and two (5%) reported access barriers in the form of registration fees (36) and access by invitation only (40). Though the majority of courses were free to audit, completion certificates often required a fee. To receive a certificate, learners typically had to pass a number of assignments. Four MOOCs (10%) offered CPD credits and 18 (45%) offered some kind of completion certificate.

Overall, the time investment for learners varied greatly, whilst one MOOC comprised a single module that could be completed in 1 h (17), others included up to 13 modules to be taken over 16 weeks (61). Direct comparisons of time investment could not be made due to differences in reported details.

### LMICs in Which MOOCs for HCWs' Education Are Accessed

**Figure 2:**
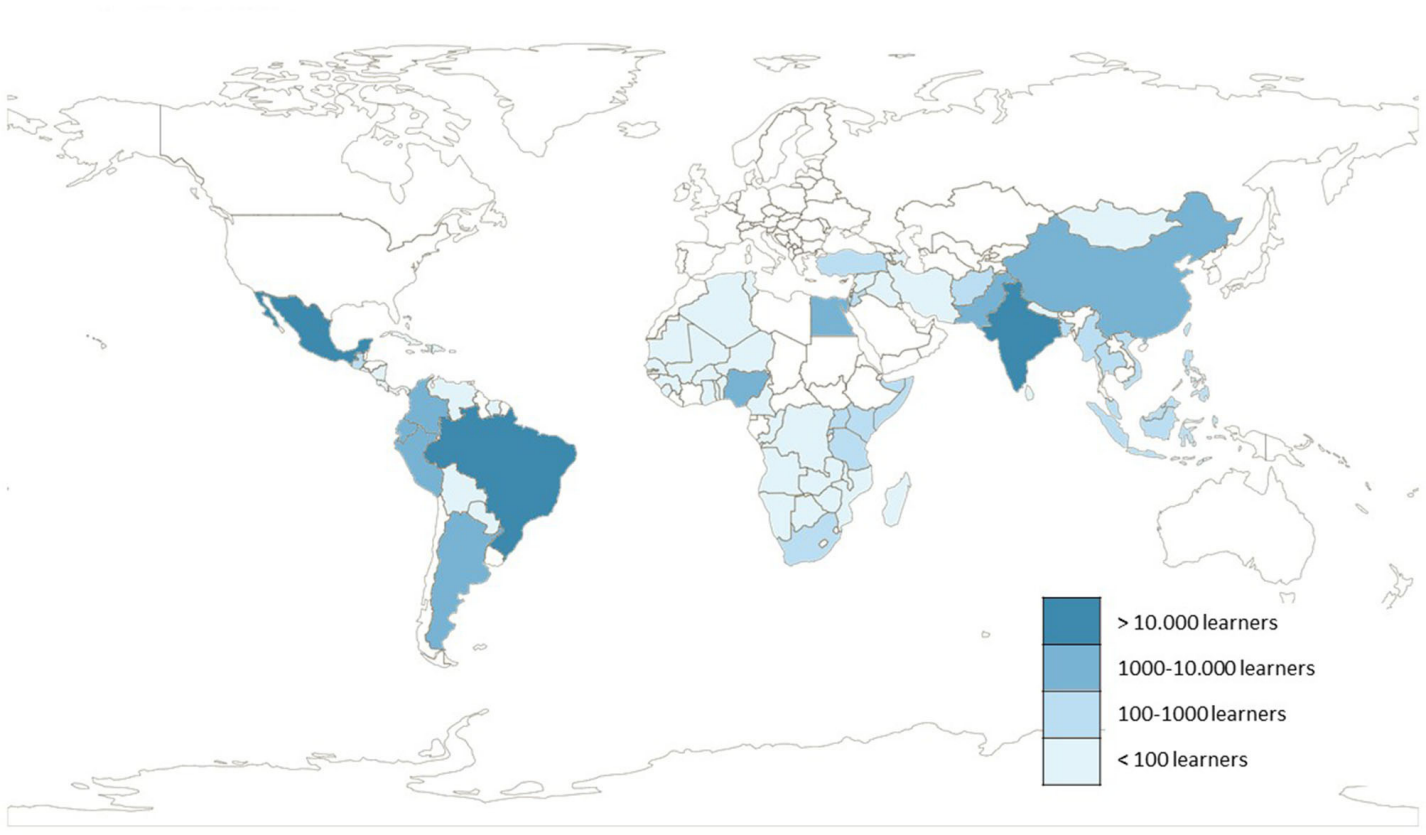
Map of LMICs Learners' location. Includes data from 28 included articles.

**Figure 3:**
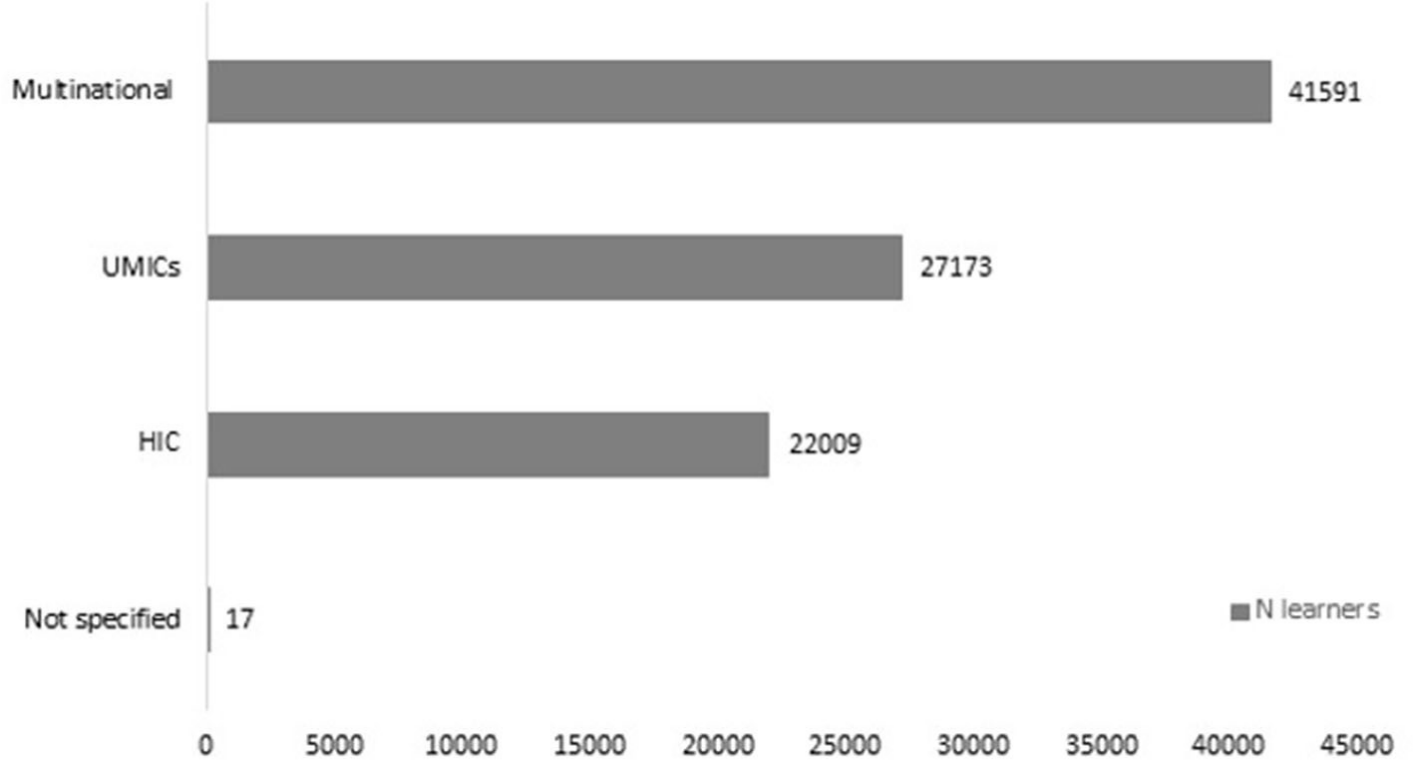
Number of learners in MOOCs from different income groups.

Documentation of learners' location varied. Eleven articles (28.2%) reported the location of all learners and 17 (43.58%) reported the number of learners from the top participating countries only. Included MOOCs were accessed in 98 LMICs (see Figure 2), of these 21 were LICs, 32 lower-middle-income countries, and 36 upper-middle income countries. The countries with the highest representation of learners were Mexico (*n* = 57,248 learners across all included MOOCs), Brazil (*n* = 16,743 learners across all included MOOCs), and India (*n* = 11,294 learners across all included MOOCs). Although most of the MOOCs accessed by learners from LMICs are developed in HICs (see Table 3), there are more learners from LMICs attending MOOCs developed by a multinational team or even upper-middle income countries than HICs (Figure 3).

### HCWs Targeted by MOOCs

The majority of MOOCs (*n* = 22, 56%) were aimed at postgraduates and although no pre-requisite knowledge on the topic was required, some knowledge or experience in the broader field was assumed. For example, the MOOC on physiotherapy for spinal cord management did not assume any knowledge of spinal cord treatment but targeted learners with physiotherapy experience (39, 70, 71). In addition to the MOOCs for postgraduates, nine (22%) were directed toward HCWs in training, and nine (22%) were aimed at the general public whilst relevant to HCWs. Overall, MOOCs targeted a wide range of HCWs including nurses, midwives, dentists, psychologists, and physicians to all those working in the broader health care sector. Five (7.5%) MOOCs were HCW unspecific, targeting those interested or involved in the topic (36, 37, 49, 64). For example, a MOOC on breast cancer detection targeted health promoters, nurses, general physicians, and medical students (64), and a MOOC on antimicrobial resistance targeted physicians of different specialties, nurses, and biomedical and clinical scientists (36). Fricton et al. (49) found it difficult to strike a balance acceptable for such a diverse audience and Medina-Presentado et al. (36) reported that the nurses in their course felt that the assessments did not benefit them as they focused on medication prescription, which they were not accredited for.

### Health-Related Topics Covered by MOOCs

Twenty-one MOOCs (52.5%) focused on specific diseases or injuries and their treatment. Of these, 11 (27.5%) focused on infectious diseases and were predominantly developed as part of an epidemic response (18, 38, 50, 65), eight (20%) focused on the treatment and prevention of non-communicable diseases, including diabetes (68), chronic pain (49), undernutrition (51), dentistry (58), and dementia (46, 72), and two (5%) on the treatment of injuries (39, 57). Communication techniques and patient interaction was the focus of five MOOCs (12.5%) (43, 55, 59, 60, 66), four (10%) focused on research practice, three (7.5%) on responding to emergencies (37, 61, 62), and two (5%) on evaluating and managing quality care (40, 52). Seven MOOCs (17.5%) could not be categorized, as these covered the topics of global health (67) and global health experiences (41), climate change and health (42), teacher training (47), neurobiology (63), and health informatics (56) (see Table 5 for an overview).

**Table 5:** Facilitators and barriers of learning by MOOC.

**Factor**	**Facilitator**	**Barrier**
**Social interaction**	Tutors in discussion forum Availability of local facilitator Blended learning options Interaction with other learners	Interaction with other learners Blended learning option
**Academic skills**	Availability of multiple language options	MOOC not provided in local language
**Content related issues**	Part of epidemic or pandemic response Integrated into local focus	
**Technical skills and problems**	Functional and easy to handle instructional design	Internet connectivity Digital literacy
**Situational issues**	Support from government and employers: involvement and promotion Part of a university degree	Time: deadlines to short, balance learning with work and family obligations
**Individual motivation**	Regular deadlines Email reminders	Voluntary nature of course

We analyzed the MOOC learning objectives according to their level of complexity and specificity by extracting the action verbs using Bloom's taxonomy as a framework (73). To do this we extracted the action verb and assigned it to a level within the framework using the “Pragmatic Master List of Action Verbs for Bloom's Taxonomy” (74) as guidance.

**Figure 4:**
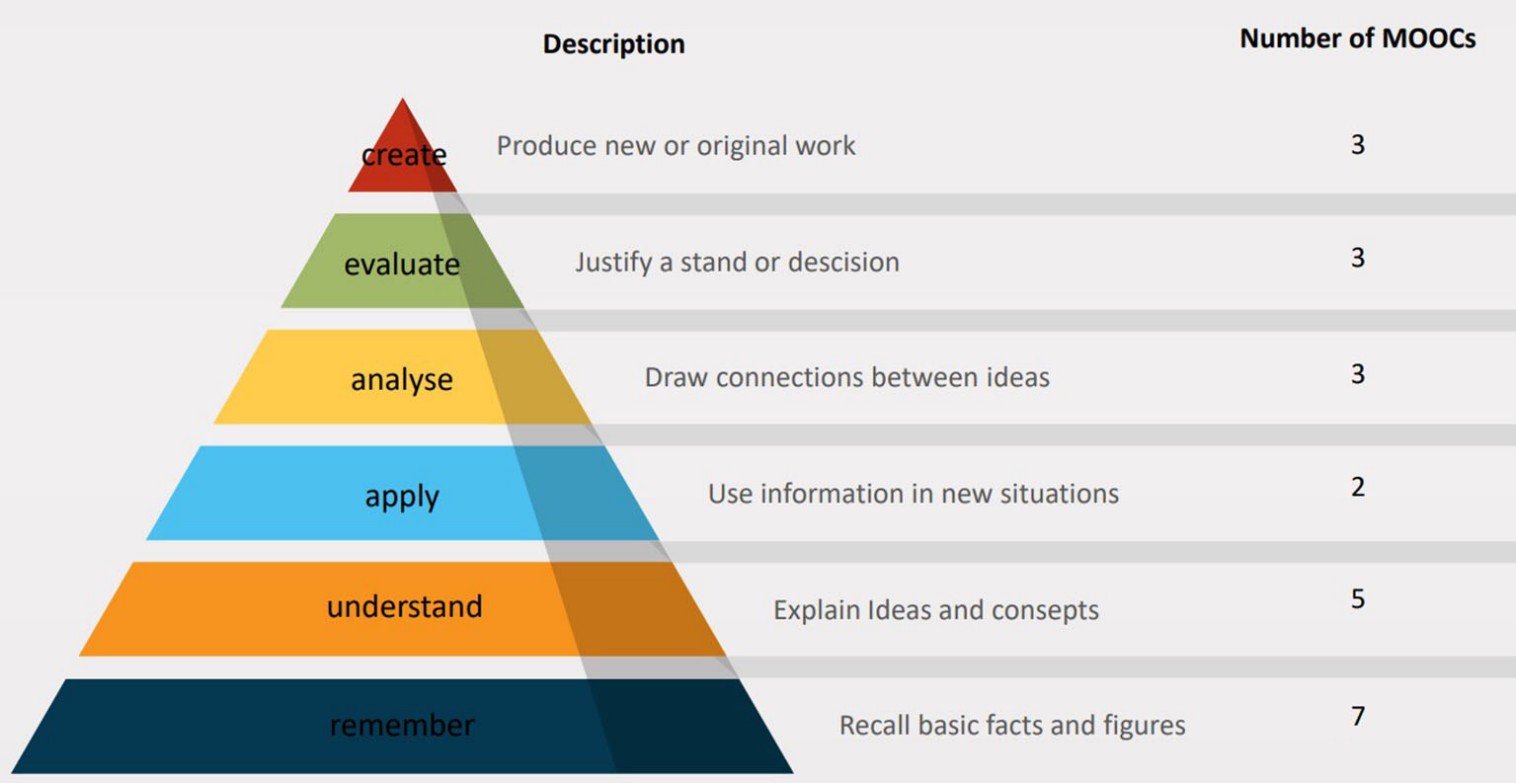
Level of learning across 10 MOOCs according to Blooms' taxonomy.

This analysis was limited by the fact that only 11 (27.5%) articles reported the learning objectives. Within these, 10 (25%) MOOCs included objectives associated with knowledge transfer, that is remembering (37, 39, 40, 45, 49, 52, 64, 70) (level 1) and understanding (18, 45, 54, 56, 64) (level 2) concepts, ideas, and treatments. Five MOOCs (12.5%) included applying (44, 64) (level 3), analyzing (44, 52, 56) (level 4), evaluating, justifying (40, 44, 54) (level 5), or creating (40, 44, 70) (level 6) treatment plans or research (see Figure 4 for an overview).

### Facilitators and Barriers Described by MOOC Learners in LMICs

Facilitators and barriers to using MOOCs were reported in 11 studies (27.5%) and were identified through online post-course surveys, discussion groups, and completion rates, course uptake, and reported satisfaction. Of these 11 studies, nine (22.5%) specified those for learners from LMICs, and are the ones reported here. We used the categories developed by Henderikx et al. (75, 76) to structure facilitators and barriers into six groups: *social interaction, academic skills, content-related issues, technical factors, situational issues*, and *individual motivation* (see Table 6).

**Table 6:** Overview of the health-related content areas covered by the included MOOCs.

**Subject/topical category**	**No. of MOOCs (%)**	**References**
Disease and injuries	21 (52.5%)	
Infectious diseases	113 (27.5%)	(17, 18, 36, 38, 45, 48, 50, 54, 65, 70, 75)
Non-communicable diseases	8 (20%)	(46, 49, 51, 53, 58, 64, 68, 72)
Injuries	2 (5%)	(39, 57)
Communication	5 (12.5%)	(43, 55, 59, 60, 66)
Research training	2 (5%)	(44)
Emergency treatment	3 (7.5%)	(37, 61, 62)
Quality of care	2 (5%)	(40, 52)
Other topics	7 (17.5)	(41, 42, 47, 56, 63, 67)

Social interaction with other learners was identified both as a barrier and a facilitator. Some learners indicated that they enjoy, profit from, and seek (70, 71) interaction with other learners, others perceived it to be a burden and reported it as their least favorite part of the course (36, 70). In addition, interaction with facilitators (40, 44) and the availability of a local facilitator (52) assisted course completion. Likewise, blended learning options that combined online phases with face-to-face sessions, in which participants living in the same region had the opportunity to meet regularly to discuss the content, were identified as factors that promoted retention and course completion (52, 61, 64, 67). Warugaba et al. (67) found that the number of in-person sessions attended was correlated with successful MOOC completion and Jia et al. (61) found that blended learners learned more when compared to “social learners.”

Academic skills in terms of difficulty were not mentioned by HCWs, but language was reported as a barrier by Scott et al. (52) as the MOOC was not available in the local languages French or Kinyarwanda (mainly spoken in Rwanda). Similarly, some studies assumed the availability of different languages facilitated course uptake (36, 38) based on increased uptake after the addition of different languages (18).

Course update was especially rapid in MOOCs established as part of an epidemic response, such as the MOOC developed in response to the Ebola outbreak in Africa as reported by Evans et al. (50) and the MOOC on Cholera contamination (65). In both cases, the MOOCs were perceived to be very “useful” (50) and “educational” (65) in the affected regions. Similarly, integration into ongoing local initiatives was reported to facilitate uptake (52).

Technical problems in terms of internet problems including fluctuating internet connection were frequently mentioned (38, 40, 52, 67). Providing access to course media such as video content in lower resolution and downloading course materials for offline usage in places with a better connection were reported as viable solutions to this problem (52, 67). Digital skills were reported as an issue for older learners who lacked digital literacy by Magaña-Valladares et al. (65). This study also reported that the issue could be overcome by face-to-face peer support. On the other hand, a functional and easy to handle instructional design that facilitated learning (65) was reported as a facilitator to learning.

The endorsement of the course by employers and the local government through involvement and promotion was reported as a facilitator (52, 64, 65), as was the promotion of courses through trusted sources such as UNICEF (51) or professional networks (36, 39, 51). Furthermore, the learners' individual situation was mentioned as a barrier in terms of time availability caused by short deadlines (40) or conflicts of priorities between work, family responsibilities, and learning (36, 40, 52). In contrast to this, regular deadlines and email reminders (39, 51, 52) were reported to facilitate timely completion of the course. Finally, a single study reported that the voluntary nature of the course was a barrier to learners (52).

### MOOC Evaluation Criteria

We identified a total of six criteria that were used to describe success in the included studies: completion rate, reach, learner evaluation, attitude, knowledge, and action (Table 7 for an overview).

**Table 7:** Six criteria used to define success of MOOCs identified in included studies^*^.

**Evaluation criteria**	**No. of MOOCs (%)**	**Outcomes as reported in the studies**	**Reference**
Completion rate	28 (70%)	Mean = 32.62 SD = 28.04 Ranged: 1%−100%	(38–43, 47–56, 58, 60, 61, 64, 65, 67, 70, 72)
Reach	5 (12.5%)	Reached targeted audience, including learners that may otherwise not have had access to educational resources as offered by the MOOC	(17, 18, 38, 57, 65)
Learner evaluation**	21 (52.5%)		
*Course rating out of five*	3 (7.5%)	In all cases ratings were above 4/5	(50, 53, 57)
*Survey responses*	13 (32.5%)	Overall high level of satisfaction, achievement of personal learning objectives	(36, 38–40, 49–51, 56, 59, 68, 71)
*Written or reported comments on the courses*	7 (17.5%)		(36, 49, 53, 55, 65)
Attitude	1 (2.5%)	No significant change in attitude	(46)
Knowledge	30 (75%)		
*Pre-post*	7 (17.5%)	All studies reported a significant improvement in test scores	(36, 38–41, 52, 59, 70, 71)
*Post*	6 (15%)		(53, 57, 60, 65, 67, 68)
*Compared to other teaching modalities*	3 (7.5%)	Learning with the support of MOOCs was equally good if not better than compared with other modalities	(61, 62, 71)
Action	9 (22.5%)		
*Intentions*	7 (17.5%)	Expected impacts or intentions to implement learned content reported by the majority of respondents	(49, 51, 57, 59, 67, 70, 71)
*Behavior*	2 (5%)	Changes were reported by less than half of the respondents	(37, 48)

* Based on n = 40 MOOCs, **Articles may have reported on several sub-categories of learner evaluation.

Success of the MOOC in terms of reaching a broad audience and remote learners was not predefined but reported for five (7.5%) MOOCs, all of which concluded success based on reaching a diverse audience including learners from remote locations.

Completion rate was reported for 28 MOOCs (70%) and ranged greatly from 1 (42) to 100% (71) (Mean = 32.62, SD = 28.04). However, definitions of completion varied: one reported the number of active learners (68), some reported the number receiving a certificate or completing the final module (52), and others used Google Analytics (39, 70) as a point of reference. Completion rate was considered successful in some cases when retention was higher than the 10% typically reported in the literature (50) and was usually highest in those courses that were embedded or promoted in a formal structure such as a university course or blended learning (52, 61, 64) and lowest when there were few promotional efforts (42). However, there were exceptions to this rule for example the MOOC on understanding dementia had a completion rate of 42% without reporting promotional efforts (72).

Acceptance and learner evaluations were reported in 22 studies (56.41%). In post-course evaluations, mostly questionnaires, learners provided feedback (*n* = 7, 17.9%), rated the MOOC (*n* = 3, 7.79%), or answered statements about the MOOC on a Likert scale (*n* = 14, 36%). In some studies, learners suggested changes, but overall, feedback was predominantly positive (50, 53, 57), with learners reporting high levels of satisfaction and usefulness, as well as achievement of personal learning goals. Chan et al. (37) collected feedback from dropouts and found time management issues rather than disliking the course to be the predominant reason.

An assessment of knowledge in the form of regular quizzes or final assessments was included in 30 MOOCs (75%). The results of these assessments were reported in 15 studies (38.5%), whereby nine (23%) included pre- and post-knowledge assessments that showed an increase in knowledge for all studies (36, 38–41, 52, 59, 71). Six studies (15.4%) included post-MOOC assessments (53, 57, 60, 64, 67, 68) and four studies (9.75%) compared MOOC learning to other teaching modalities such as self-regulated online learning (71), face-to-face learning (62) supported by MOOCs (63) or MOOCs embedded in a blended format (61).

One study looked at attitude and found that learners' attitude toward dementia, the topic of the MOOC, did not significantly change after the course (46).

The impact of MOOCs on HCWs' behavior and patient care was investigated by nine studies using quantitative measures (23%). Of these, seven (18%) reported on impact and behavior change collected in post-course surveys. For example, learners participating in a MOOC on diabetes reported having learned something they felt they could implement at work (68). Fricton et al. (49) observed that 85% (*n* = 300) of HCWs taking the MOOC on managing chronic pain intended to modify their patient care. A follow-up investigation of self-reported behavior change was reported by two studies (5%) (36, 48). Both found that less than half of those study participants responding had acted on commitments to change (36, 48).

## Discussion

We found 40 MOOCs relevant to HCWs education that were accessed by learners in over 90 LMICs. and although overall feedback of study participants was positive, some regions and countries such as Central Africa were not represented, and key causes of diseases were not addressed. Most importantly we found that we were often unable to distinguish between outcomes by cohort due to inconsistent reporting of MOOC characteristics which restricted summarizing of results and conclusions.

MOOCs covered a wide range of health-related content over several health disciplines, with the diagnosis and treatment of diseases, particularly infectious diseases, being the most prevalent and their use in disseminating information to HCWs as part of an epidemic or pandemic response being notable. Communication and patient interaction learning aims were incorporated in a few MOOCs, which has been found to be lacking in medical education in some African countries (77). However, we removed articles on other topics such as palliative care (78). Other reviews on MOOCs for HCWs education have retrieved more studies (13, 20) indicating that many courses are not accessed by learners from LMICs. Furthermore, other relevant topics such as cardiovascular disease, maternal and neonatal diseases, or HIV (79) were not yet covered by included MOOCs, suggesting a possible discrepancy that should be explored. In the case of HIV, significant learning needs for HCWs have been reported (80) and it would be of interest why MOOCs are not used to fill this learning gap. Such research may also expand understanding on the limitations on the use of MOOCs for HCWs in LMICs. In addition to covering a broad range of topics, included MOOCs also targeted a broad range of HCWs, and in many cases were not focused on HCWs specifically. This could imply that MOOCs enable interprofessional education and potentially encourage interprofessional cooperation and communication, which could lead to improved patient care (81).

The learning objectives of included MOOCs centered mostly on remembering and understanding, referring to low levels of Bloom's taxonomy (73). This seems to indicate that MOOCs appear to be better suited to encourage knowledge transfer and comprehension (72). Furthermore, it may be that some MOOCs “provide more sound pedagogy that develops higher order thinking, whereas others do not” (82). Bali (82) also argues that the benefit of MOOCs may lie in the engagement between learners made possible through the course. This notion is supported by our finding that some learners reported the exchange with other learners to be particularly helpful (70, 71). Two studies implemented a “flexible pathway” of their MOOC within a blended learning environment, in which the MOOCs taught on theoretical aspects and in-person workshops were used to practice application of the learned material (64) or discuss possibilities for local implementation (52). Blended learning as a teaching and learning format is showing promising results in medical education regarding learner satisfaction and learning gain (83). Another engaging, interactive method are “virtual patients,” which train clinical decision-making skills on simulated patient cases (54, 55). More research is needed to better understand how MOOCs might be used to support skills training for HCWs in LMICs, possibly in combination with other digital learning tools such as virtual reality scenarios using head-mounted devices (84).

We found that the literature does not distinguish facilitators and barriers by learner cohort but rather reports on generic barriers reported by all included learners. Nonetheless, because the majority of included studies reporting on facilitators and barriers were targeted at specific regions and only included learners from LMICs, we can be sure that those identified are experienced by learners from LMICs. However, this means that the identified barriers are likely biased toward MOOC implementations that have taken particular precautions to facilitate uptake in LMICs. Future research should therefore focus on regional barriers experienced by MOOCs with a global audience. Despite these limitations, we found that the facilitators and barriers we identified for learners in LMICs can be compared to those reported elsewhere. Time and internet connection were the main barriers to learning with a MOOC (75, 85). Time constraints related to competing obligations around work, family life, and learning were concerns for some learners (36, 40, 52). Deadlines for assignments (43, 51) and teaching and learning modes such as blended learning approaches (52, 67) were mentioned as effective strategies for overcoming these challenges. Goal setting and contextual structuring, such as deadlines and timeframes, are likely to assist online self-regulated learning (OSRL). This is in line with previous research that has found that OSRL through goal setting is a frequently used strategy in MOOC learning and that students with high OSRL report higher satisfaction (86, 87). Limited internet connection is one of the most widely cited issues related to MOOCs and e-learning (20, 75), and are particularly relevant in the context of LMICs. Internet access continues to be a central issue, particularly in African countries where access is often still comparably expensive (10) and may be a reason for the lack of utilization in central sub-Saharan African countries. Therefore, it is essential to assure that HCWs in LMICs have access to online training materials, either by providing alternative internet access options or by providing course content offline as well, so learners can synchronize content once and then learn offline. The importance of developing an interface that is intuitive for users is highlighted by a study finding that digital literacy was a barrier whilst functional and easy to handle instructional design was identified as a facilitator (65). No barriers in terms of the content being irrelevant or too difficult were mentioned by HCWs, though nurses in an interprofessional MOOC mentioned that not all assessed content applied to them (36). On the other hand, local relevance and integration into a pan- or epidemic response were reported as factors that promote MOOC uptake. This is unsurprising and in line with the Technology Acceptance Model (88), which postulates that technology must have local relevance to be adopted. Relatedly, government and employer endorsement were concluded to be facilitators which is in line with previous research identifying lack of perceived employer support as a barrier to uptake (76). In addition, local promotion may increase awareness for MOOCs amongst potential learners leading to increased uptake, as awareness is a significant factor and has been identified as a key reason for not learning by MOOC (85).

Finally, although the language people learn in greatly impacts learning ease (89), language was not discussed much within the included articles. Further research on the extent to which language is a factor in learning through MOOCs may be useful (90) to support the choices in language options made by MOOC developers.

E-learning has been introduced in many African countries to improve HCW education (91). The fact that MOOCs are currently readily available, typically free, and often appropriate for use in diverse countries makes them a strong contender for inclusion in HCW strengthening strategies. However, the findings of our review suggest that simply the availability of MOOCs is not sufficient, rather to achieve optimal effect, they should be integrated into existing educational structures and endorsed by stakeholders and top management. For example, MOOCs could be formally included in curricula as a partial requirement, this would strengthen MOOCs' credibility in the respective countries as a serious learning and teaching tool (91). Overall, it is of note that only 11 of the included studies reported on facilitators and barriers faced by learners; also these facilitators were not formally assessed or evaluated in the included studies. Instead, they were deduced by respective authors based on experience. Although this generates valuable insights, formal research in this area would be necessary to make these findings more credible. Although barriers are more formally investigated through post course surveys, studies rarely use empirical approaches to assess barriers. A better understanding of factors that support online MOOC learners in LMICs is needed to identify how MOOCs may align with national education and training strategies for HCWs' education.

The evaluations were overwhelmingly positive and though only a few reported on barriers to taking the MOOC, there were no reports of the MOOC failing. This may be due to a response bias of learners with a positive experience being more likely to complete the evaluation (92) or publication bias of interventions evaluated as successful (93). A key finding within the evaluations is that only one study (68) reported on cohort specific evaluations indicating that learners from developing countries experienced a higher benefit from the MOOC compared to learners from developed countries. Thus the available data does not allow us to conclude whether learners in LMICs achieve similar positive outcomes as the average learners. As learners from HICs often make out the greater share, it is possible that greater usefulness or even unsuccessful participation for a sub-cohort was missed. Furthermore, included evaluations, focused mainly on short term impacts, such as learner satisfaction and knowledge gain. However, as theoretical knowledge and understanding of a procedure are not always sufficient to ensure changes in clinical practice and patient care (90, 91), these evaluations do not allow for deductions of MOOCs effectiveness in improving the quality of care. Nor do they show whether gained understanding can be retained over time.

We identified two key factors in the available literature that limited the extent to which conclusions can be drawn and summarized for our target cohort learners in LMICs. Firstly, only few articles described outcomes such as facilitators and barriers or evaluation criteria by cohort. This is likely a result of the inclusive nature of MOOCs, however, by only reporting on overall findings, conclusions for specific cohorts cannot be drawn making it difficult to suggest this teaching method to specific cohorts. Secondly, we found large heterogeneity in what MOOC characteristics the included articles reported on which impacts the extent to which conclusions can be drawn and generalized. For example, learning objectives were only reported for 25% of the MOOCs; the geographical location of learners was reported by 28% of included studies, and the delivery language was reported for 45% of MOOCs. The development and use of reporting guidelines for MOOC-based research, or research on e-learning in general, could foster progress in this field. Based on this review, the reporting guidelines for mobile health interventions (94) and the guidelines for reporting evidence-based practice education and teaching (95), we suggest that evaluations should include basic characteristics of the MOOC such as the institution or company who developed the MOOC, the teaching language including subtitles, the target audience, and learning objectives (also in respect to Bloom's taxonomy), as well as basic demographic characteristics of all learners to enable synthesis research to summaries which populations may benefit most from which characteristics. Finally, reports should include details on their evaluation (method, duration, objective, sample size, outcomes) and success criteria (rationale, evaluation methods of measuring success) where appropriate.

### Limitations

Although the search strategy was comprehensive, it is always possible that relevant studies were missed. For example, any barriers that are relevant for learners from LMICs generally but were identified in MOOCs not relevant to HCWs will have been missed as will those identified in studies addressing barriers to MOOCs without studying a MOOC. We aimed to overcome this barrier by comparing the barriers we identified to those found in the broader scientific literature. Moreover, due to our own capabilities, we were only able to include studies published in English, although this is the *lingua franca* in research (96), some articles written in local languages may have been excluded. Additionally, we did not search country-specific databases; therefore, it is possible that organizations that have published reports of interventions used at a local level, were missed. It must be kept in mind that in line with the methodological framework of a scoping review, no quality checks were conducted which may have impacted the quality of our reported findings. However, in using this methodology we were able to capture a broad range of studies including those that might otherwise have been missed (23).

Furthermore, we were only able to analyze data from MOOCs where evaluations have been published; thus, MOOCs and learner experiences that have not been published are not reflected in this review. The reporting of basic characteristics between studies is very heterogenous, oftentimes conclusions of included studies were based on a small number of studies. Many evaluations only reported on the location of learners from the most represented countries causing many countries with fewer participating learners not to be mentioned and their experience to be lost. Finally, we were not always able to distinguish between the feedback given by learners from LMICs to those from HICs which may have skewed the data.

## Conclusion

We found that MOOCs relevant to HCWs education are accessed by learners from over 90 LMICs and that these are evaluated positively in terms of learner satisfaction and learning gain for all learners in these courses. Insufficient internet connectivity and time constraints were the most commonly reported barriers, whilst support from the government and employer as well as promotion through academic sources were identified as facilitators. Included MOOCs covered a range of topics, with management and treatment of infectious diseases being the most common. However, we also identified two key limitations within the literature. Firstly, the large amount of heterogeneity between the characteristics that are reported on limits our analysis. Secondly, as only few studies reported on cohort specific findings, we were limited in the extent to which we could draw cohort specific conclusions. Therefore, we suggest the development and application of reporting guidelines for future studies.

Finally, we identified gaps in the literature including a lack of research on the actual impact of MOOCs on HCWs' practice and the subsequent benefits for patients and a mismatch between the topics covered by MOOCs and the largest burden of diseases present in LMICs.

Our findings suggest that MOOCs constitute a supplementary tool to strengthen the quality and coverage of HCW education in LMICs. While current MOOCs primarily focus on lower levels of learning (remembering and understanding) future MOOCs could strive to build on this with higher levels of learning (apply, analyze, evaluate, create).

## Data Availability Statement

The original contributions presented in the study are included in the article/Supplementary Material, further inquiries can be directed to the corresponding author.

## Author Contributions

The objective and research question were developed by JN, PN, and SB. The first round of data screening was conducted by JN and PN. The second round was conducted by JN. Data extraction, analysis, and interpretation were conducted predominantly by JN with support from PN and SB. JN wrote the manuscript draft. SB, PN, and RS all contributed to further manuscript revision. The final version of the manuscript was approved by all authors.

## Funding

This study was part of a research unit funded by the German Research Foundation (DFG). The DFG supported this research but was not involved in study design, collection, management, analysis or interpretation of data, neither in the writing of this report or in any decision to submit this report for publication.

## Conflict of Interest

The authors declare that the research was conducted in the absence of any commercial or financial relationships that could be construed as a potential conflict of interest.

## Publisher's Note

All claims expressed in this article are solely those of the authors and do not necessarily represent those of their affiliated organizations, or those of the publisher, the editors and the reviewers. Any product that may be evaluated in this article, or claim that may be made by its manufacturer, is not guaranteed or endorsed by the publisher.

## Abbreviations

CPD: continued professional development
DALYs: disability adjusted life years
e-learning: electronic learning
HCW: Health Care Worker
HICs: High Income County
LMICs: low- and middle-income country
MOOCs: Massive Open Online Courses.
