# Supplementary material for: Massive Open Online Courses for Health Worker Education in Low- and Middle-Income Countries: A Scoping Review
Source: Front Public Health. 2022 Jul 12;10:891987. doi: 10.3389/fpubh.2022.891987 (PMC9315291; doi:10.3389/fpubh.2022.891987)
Supplement: Supplementary file 1 [file Table_1.DOCX]

# Appendix 1

## Search Strings

| **Journal** | **Search String** |
| --- | --- |
| Scopus | TITLE-ABS-KEY ( mooc* OR "Massive Open Online Course" ) AND TITLE-ABS-KEY ( health* OR "public health" OR healthcare OR "health professional" OR "health care worker" OR "health personnel" OR "allied health personnel" OR "human resources for health" OR "health care provider" OR "health occupation" OR "allied health occupation" OR nurse* OR doctor* OR midwife OR dietician* OR "medical education" OR "health education" OR "medical student" OR "Allied health occupation" OR “community health worker” ) |
| Web of Science | #1 TS=(MOOC OR “Massive Open Online Course”  #2 TS=( health* OR "public health" OR healthcare OR "health professional" OR "health care worker" OR "health personnel" OR "allied health personnel" OR "human resources for health" OR "health care provider" OR "health occupation" OR "allied health occupation" OR nurse* OR doctor* OR midwife OR dietician* OR "medical education" OR "health education" OR "medical student" OR "Allied healthboccupation" OR “community health worker”  #3 combine sets with AND: #1 AND #2 |
| PubMed | (MOOC*[Title/Abstract] OR "massive open online course*"[Title/Abstract]) AND ("Health Personnel"[MeSH Terms] OR "Allied Health Personnel"[MeSH Terms] OR "Nurses"[MeSH Terms] OR "Health Occupations"[MeSH Terms] OR "delivery of health care"[MeSH Terms] OR "health*"[Title/Abstract] OR "public health"[Title/Abstract] OR "healthcare"[Title/Abstract] OR "health professional*"[Title/Abstract] OR "health care worker"[Title/Abstract] OR "health personnel"[Title/Abstract] OR "allied health personnel"[Title/Abstract] OR "human resources for health"[Title/Abstract] OR "health care provider*"[Title/Abstract] OR "health occupation"[Title/Abstract] OR "Allied health occupation"[Title/Abstract] OR "nurse*"[Title/Abstract] OR "doctor*"[Title/Abstract] OR "midwife"[Title/Abstract] OR "dietician*"[Title/Abstract] OR "medical education"[Title/Abstract] OR "Health education"[Title/Abstract] OR "medical student*"[Title/Abstract] OR "Allied health occupation"[Title/Abstract] OR “community health worker"[Title/Abstract]) |
| ERIC | 1 health.mp or exp Health/  2 public health  3 healthcare  4 health professional*.mp.  5 health care worker.mp.  6 health personnel.mp. OR exp. Health Personnel/  7 allied health personnel.mp. OR exp Allied Health Personnel/  8 human resources for health.mp  9 health care provider*.mp  10 health occupation.mp =R exp Health Occupations/  11 allied health occupations.mp or exp Allied Health Occupations  12 exp Nurses/ or nurse*.mp.  13 exp Physicians/ or doctor*.mp  14 midwife-mp  15 exp Dietetics/ or dietician*.mp.  16 medical education.mp. or exp Medical Education/  17 Health education.mp or exp Health Education/  18 exp Medical Students/ or medical student*.mp  19 “Community Health Services AND/OR Health Personnel”/  20 MOOC.mp or exp Online Courses/  21 massive open online course.mp  22 1 or 2 or 3 or 4 or 5 or 6 or 7 or 8 or 9 or 10 or 11 or 12 or 13 or 14 or 15 or 16 or 17 or 19  23 20 or 21  24 22 and 23 |
| Google Scholar | (mooc OR "massive open online course") AND (“heath personnel” OR “health care worker” OR “allied health occupation” OR “community health worker” OR “medical staff” OR “health professional” OR “health care provider” OR healthcare OR “medical education” OR “health education”) |
