# Supplementary material for: Massive Open Online Courses for Health Worker Education in Low- and Middle-Income Countries: A Scoping Review
Source: Front Public Health. 2022 Jul 12;10:891987. doi: 10.3389/fpubh.2022.891987 (PMC9315291; doi:10.3389/fpubh.2022.891987)
Supplement: Supplementary file 2 [file Table_2.docx]

# Appendix 2

## Summary of charted data

| **Author, Date** | **MOOC characteristics** (1. MOOC origin; 2. Hosting platform; 3. Language & subtitles; 4. Certificate, 5. Teaching format) | **Topic and level of learning objectives according to Blooms taxonomy** | **LMICs participants** | **Barriers/facilitators** | **Evaluation** |
| --- | --- | --- | --- | --- | --- |
| Garrido, 2017 | 1. Brazil  2. UNA-SUS (other)  3. na/na  4. continued dental education | Issues surrounding dental care for people with diabetes, hypertension, and chronic kidney disease | All 13089 from Brazil | na | 10 MCQ questions, results are not reported  29% completion rate |
| Costa et al., 2020 | 1. Europe  2. Canvas  3. English, no subtitles  4. certificate of completion  5. chapters unlocked weekly, self-regulated learning | CBT techniques for psychological treatment | Turkey: 243, Egypt: 227, Russia: 327 | na | 13% completion rate |
| Fife, 2016 | 1. South Africa  2. Future Learn  3. na/na  4. na | Humanising Health care: communication | 700 from South Africa |  | na |
| Clark, 2017 | 1. UK  2. na  3. na/na | Fungal infections: How to collect, process, and analyse specimens for fungal infections  Level 1 and 2 learning objectives | Learners from Brazil, Chile, Kenya, Senegal, Uganda, Indonesia, Vietnam, Los, Thailand Malaysia, Kuwait, Pakistan |  |  |
| Rodrigues & Leinster, 2016 | 1. UK  2. Future Learn  3. na/na  4. na | Clinical Supervision | Learners from several African countries, no specific numbers provided |  | 37% completed the course, 52% rated overall experience as excellent in post-course survey |
| Dalsgaard, et al., 2019 | 1. Denmark & Australia  2. other  3. na/na  4. certificate of completion | Introduction to evidence-based practice  Levels 3, 4, 5, and 6 learning objectives | Learners from Africa, Bhutan, Myanmar, Nepal, Papua New Guinea, South Sudan | **Facilitator**: availability of facilitators |  |
| Borges et al, 2017 | 1. Brazil  2. Future Learn  3. na/na  4. na  5. developed as part of university degree for nursing, physiotherapy, and medicine students | How to conduct patient home visits | All 135 participants from Brazil |  | Pre and post course knowledge test: significant increase, self-reported perceived ability improved, educational strategies and time largely evaluated as (very) adequate in past course survey |
| Robershaw et al., 2019 | 1. UK  2. na  3. na/na  4. na | Dementia as a disease that changes over time | 3 learners from Africa |  | Non-significant improvement in attitude towards dementia and dementia patients |
| Eccelstone, 2019 | 1. Australia  2. na  3. na/na  4. na | Basic neurobiology of dementia, its pathophysiology, medical management, and person-centred care | 244 from the Philippines, 294 from India, 147 from China, 295 from Malaysia |  | Pre-post course knowledge assessment. Learning gain greatest for those with no prior experience.  Completion rate: 42% |
| Fricton et al 2015 | 1. USA  2. Coursera  3. English/English  4. na | Human systems approach to chronic pain | 25934 (25%) learners from emerging and developing countries:  India: 1.236, China: 741, Brazil: 494, Russia: 247 |  | Knowledge assessed in course but results not reported, high learner satisfaction: relevant and applicable. 85% indicated changes in patient care. 8% completed quizzes for each module |
| Hooley et al., 2020 | 1. WHO, USA, Rwanda  2. na  3. subtitles in English, French, and Spanish  4. participation certificate | Challenges of different health care settings, assessing, developing, and implementing disease control strategies Designing research around interventions.  Level 1, 5, and 6 learning objectives | 92 learners from Rwanda | **Barriers**: Internet connectivity, time commitment due to conflict of priorities and tight deadlines. The final report was challenging due to lack of experience.  **Facilitators**: Additional support by the research team: initial meeting plus Q&A sessions | Knowledge assessed in course but results not reported. 38% completion rate (low but higher than average MOOC) |
| Stark & Pope, 2014 | 1. USA  2. Cornell nutrition works  3. na/na  4. 12 CME credits | Undernutrition in infant and young child feeding. Introduction to interventions. Infant and young child feeding in the context of HIV | "Afghanistan: 180  Ethiopia: 300  India: 1075  Kenya: 760  Indonesia: 150  Nigeria: 175  Pakistan: 200  Somalia: 250  Tanzania: 150  Uganda: 150 | **Facilitators**: promoted through academic networks, timeline and notifications about deadlines motivated timely completion | Knowledge assessed in course but not reported, 32% completion rate, high intentions to apply what was learned assessed at 1-year follow-up |
| Evans et al., 2017 | 1. USA  2. coursera  3. English  4. certificate of participation or pass with distinction | Ebola virus: patient care; prevention, treatment and response, laboratory issues; potential and diagnosed Ebola patients; impact of Ebola on global health | 71 from Nigeria  Liberia: 14  Sierra Leone: 14  Guinea: 7 (Liberia, Sierra Leone and Guinea all at centre of epidemic)  36% (2549) from emerging economies  7% (496) from African continent. |  | Knowledge assessed in course but results not reported. 10% completion rate (noted to be higher than average).  Primarily positive feedback, course rating: 4.73/5 |
| Albrechtsen et al., 2017 | 1. USA  2. coursera  3. na/na  4. certificate available | Epidemiology, prevention of diabetes, obesity, pharmacological treatment of diabetes, genetic forms of diabetes | WHO development regions |  | Subjective knowledge gain reported (objective gain assessed but not reported9, particularly learners from developing countries felt they learned something they can use in their current job |
| Zhou et al., 2020 | 1. China  2. na  3. na/na  4. na  5. developed for medical interns | Emergency and critically ill nursing | All 30 from China |  | No differences in knowledge gain between groups, MOOC learners reported higher satisfaction |
| Koch & Hägglund, 2017 | 1. Sweeden  2. wdC  3. na/na  4. yes | eHealth. introduction to the concept, use in health system and healthcare and for HCP, designing eHealth  Level 2 and 4 learning objectives | 1290 from India | **Facilitator**: Platform that facilitates interaction | 4.41% completed at least one instance  Majority reported achieving personal learning goals |
| Sneddon et al., 2018 | 1. UK  2. future learn  3. English, subtitles in English, simplified Chinese, Latin American Spanish, and Russian  4. certificate of completing (min 90%)  a statement of participation for completing at least 50% | Introduction to antimicrobial stewardship: why should antibiotic use be measured, who is it measured?; strategies for antimicrobial stewardship | 4283 (13%) from Africa |  | Knowledge assessed in course but results not reported.  9.1% of registered users completed 1 step/week.  49% had implemented stewardship interventions at 6-month follow-up  Over 90% rated the course as good or excellent and indicated that it met or exceeded expectations |
| Chan et al., 2019 | 1. Guatemala and Spain  2. na  3. na/na  4. na | Strategies for taking care of ill patients, essential first aid, general information about allergies and poisoning, how to prevent disease and improve health outcome  Learning objective: level 1 | 138 Guatemala.  46 Colombia  33 Mexico  26 Peru |  | Knowledge assessed in course but results not reported.  2.37% completion rate  69% indicated that the course was useful and met their expectations.  Forums were not very useful |
| Harvey et al., 2014  Harvey et al., 2017 | 1. international spinal cord society  2. Physiopedia  3. English  4. certificate of completion and 15 international physical therapy CPD credits | Physiotherapy treatment of spinal cord injuries  developed in cooperation with spinal cord society  Level 1 learning objective | Algeria: 12  American Samoa: 2  Argentina: 124  Armenia: 1  Azerbaijan: 2  Bangladesh: 61  Benin: 1  Bhutan: 1  Bolivia: 1  Bosnia and Herzegovina: 5  Botswana: 3  Brazil: 394  Bulgaria: 13  Burma: 3  Burundi: 10  Cameroon: 9  Cape Verde: 6  China: 122  Colombia: 16  Congo: 4  Costa Rica: 43  Dominica: 2  Dominican Republic: 36  Ecuador: 2  Egypt: 920  El Salvador: 1  Eritrea: 1  Ethiopia: 12  Fiji: 26  Ghana: 6  Grenada: 2  Guatemala: 12  Guyana: 10  Haiti: 14  India: 2004  Indonesia: 36  Iran: 3  Iraq: 3  Jamaica: 198  Jordan: 130  Kenya: 40  Lebanon: 39  Libya: 1  Madagascar: 18  Malawi: 1  Malaysia: 426  Maldives: 3  Mali: 1  Mauritius: 7  Mexico:120  Micronesia: 1  Mongolia: 5  Mozambique:1  Myanmar: 163  Namibia: 20  Nepal :45  Netherlands Antilles: 1  Niger: 51  Nigeria: 207  Pakistan: 630  Palestinian Territory: 80  Panama: 4  Peru: 16  Philippines: 111  Romania: 9  Russian Federation:8  Rwanda: 60  Saint Lucia: 2  Saudi Arabia: 282  Serbia :5  Senegal : 1  Sierra Leone: 1  Solomon Islands: 1  Somalia : 1  South Africa: 278  South Korea: 2  Sri Lanka: 94  Sudan: 66  Suriname: 4  Swaziland: 4  Syrian Arab Republic: 5  Tanzania: 5  Thailand: 144  Togo: 3  Tunisia: 1  Turkey: 10  Tuvalu: 1  Uganda : 14  Ukraine : 22  Vanuatu: 1  Venezuel: 1  Vietnam: 105  Yemen: 6  Zambia: 45  Zimbabwe: 11 | Course was widely advertised through social media and news letters  Interaction with others through Facebook group was positively and negatively rated | Significant knowledge increase between pre-and post-course assessment.  Course met expectations |
| Houssain et al., 2015 | 1. international spinal cord society  2. Physiopedia  3. English  4. na  5. offered as part of physiotherapy degree | Physiotherapy treatment of spinal cord injuries  developed in cooperation with spinal cord society  level 1 learning objective | All 24 from Bangladesh |  | Knowledge increased as a result of MOOC participation, but no more than in self-organized online learning  100% completion rate.  Perceived confidence to treat others patients was higher in MOOC than self-organised online learners |
| Stathakarou et al., 2018 | 1. Sweeden  2. edX  3. na  4. na | Urology  Level 2 and 5 learning objectives | Some learners from India |  | 10.45% completion rate  Reached learners in remote areas |
| Barteit et al., 2019 | 1. Germany and France  2. Iversity  3. English; subtitles: Arabic, Chinese-Mandarin, English, Hindi, Indonesian, French, Portuguese, Russian, Spanish.  4. participation certificate | Climate Change and Health | India: 5.9% (161), Philippines: 3.9% (107), Nigeria: 2.2% (60), Egypt: 1.4% (38), Kenya: 1.3% (35) |  | Voluntary knowledge assessment at end of each module, results not reported  1% completion rate |
|  | 1. Germany, France, & Burkina Faso  2. FUN MOOC  3. French, na  4. participation certificate | Climate Change and Health with a focus on West Africa | Burkina Faso: 0.32% (8), Senegal: 0.31% (8), Algeria: 0,28% (7), Tunisia: 0,25% (6), Mali: 0.2% (5), Togo: 0,18% (4), DRC: 0.18% (4), Guinea: 0.17% (4); Ethiopia: 1% (2) |  | Voluntary knowledge assessment at end of each module, results not reported  9% completion rate |
| Canavese et al., 2019 | 1. Brazil  2. na  3. an/na  4. certificate available | LGBTQ+ health rights | All 582 learners from Brazil |  | Participants needed to pass short MCQs to progress. This means those who completed the course were able to answer all questions |
| Scott et al., 2019 | 1. USA  2. edX  3. an/na  4. certificate of completion and CPD credits  5. blended option in Rwanda | High quality health care  Level 1 and level 4 learning objectives | 396 from Rwanda enrolled, 179 (45.2%) certified.  India: 1899 registered, 116 certified  Brazil: 1084 registered, 30 certified  Mexico: 911 registered, 33 certified  Nigeria: 785, 53  Colombia: 545, 16  Egypt: 323, 15  Pakistan: 382, 19  Turkey: 368, 11  Philippines: 220, 16  China: 304, 110. | **Barriers**: limited internet coaction, time because of conflict of priorities between learning and working, language (French and Kinyarwanda not available), voluntary nature of programme  **Facilitators**: Promotion through Ministry of Health, appointment of facilitators at district level, alternatives for areas with low internet connectivity, CPD qualification, integration with local initiatives | Knowledge assessed in course but results not reported.  Completion rate given for top 20 countries. Ranged from 1.9% to 45.2%.  Helped learners identify ways of improving quality of care in their workspace |
| Fermont et al., 2020 | 1. Canada  2. Mon Portal  3. French/na  4. certificate of completion | Sports related concussions. how to avoid them and how to manage them (protocol) when they occur | 6.8% (569) from Haiti, 11.2% from Africa |  | 60% MCQs answered correctly by 26-32%. Implementation intentions reported |
| Magana-Valladares et al., 2016 | 1. Mexico  2. na  3. an/na  4. na  5. blended: MOOC + 4h face to face workshop | health promotion actions to reduce the risk of health loss in breast cancer: early detection, identification of breast abnormalities, referral process to provide adequate and timely guidance. Information on institutions and organizations that provide multidisciplinary support to the relatives of women with breast cancer.  Level 1, 2 and 3 learning objectives | All 11569 from Mexico | **Facilitators**: strong promotion through government and stakeholders, close monitoring, blended learning | Pre and post course formative and summative assessments, results not reported.  88% of those enrolling graduated |
| Berman et al., 2017 | 1. Sweeden  2. edX  3. English subtitles  4. certiciate available | Behavioural medicine | India: 1685 (8.97%); Egypt: 392 (2.09%); Brazil: 354 (1.88%); China: 288 (1.53%); Mexico: 272(1.4%); Pakistan: 223 (1.19%); Philippines: 222 (1.18%); Nigeria; 180 (<1%); Indonesia: 158 (<1%); Columbia: 142 (<1%) |  | Knowledge assessed in course but results not reported. |
| Magana-Valladares et al. 2018 | 1. Mexico  2. na  3. na  4. accreditation given if final score above 70 | Proper cholera containment and management measures: concept of cholera and prevention procedures; diagnosis; treatment; key aspects to conduct a timely epidemiological surveillance | All 35698 from Mexico | **Barrier**: lack digital literacy amongst older learners, overcome by peer support  **Facilitators**: educational design, clear messages with clear graphical content, peer support and local organisation to ensure involvement, commitment of senior management | Post-course MCQ: average score was 85.6/100, 6 did no pass. |
| Jia et al., 2019 | 1. China  2. icourse163  3. na  4. na  5. blended learning option as part of degree | Health assessment and examination | All 57 blended learners from China, location of other learners not provided |  | Blended learners had significantly better test and final assessment results than other learners. Committed other learners did equally as blended learners. 100% and 7.14% completion rate amongst blended and other learners respectively |
| France et al., 2020 | 1. USA  2. coursera  3. English  4. participation certificate available | Relationship between dental medicine and other health issues | Egypt ~13% (600)  Mexico ~3% (140)  Pakistan 3% (140)  Turkey 3% (140)  Brazil ~2% (90)  India ~10% (460) |  | Knowledge assessed in course but results not reported.  Course rating: 4.9/5 |
| Warugaba et al., 2016 | 1. Switzerland  2. coursera  3. na  4. na  5. blended learning option |  | 62 from Rwanda | **Barrier**: unstable internet access | 90% passed 5-7 quizzes and high self-reported skill increase 80% expect acquired skills to improve work. 53% successfully completed course. |
| Jacquet et al., 2018 | 1. International cooperation  2. edX  3. na  4. na  5. part of global health programme for medical students | The practitioners guide to global health  aim: prepare learners to engage in safe and ethical global health experiences  target: medical students in HIC | India: 297 (part 1), 314 (part 2), 522 (part 3) |  |  |
| Rohloff et al., 2018 | 1. WHO  2. openWHO  3. French & English  4. no | Plague | Learners from  Madagascar: 18; 26 from neighbouring: South Africa, Mozambique, Kenya, Seychelles, Mauritius, Reunion, and Ethiopia |  | satisfying outreach to targeted audience and demonstrated the ability of training a large number of people on a complex issue at a low cost |
|  | 1. WHO  2. openWHO  3. na  4. no | Diphtheria | Bangladesh: 48, Yemen: 28, India: 24 |  |  |
|  | 1. WHO  2. openWHO  3. Englsih, Frnch, Lingala  4. no | Ebola | Learners from DRC, Uganda, Zambia, Tanzania, Cameroon, Rwanda |  |  |
| Utunen et al., 2020 | 1. WHO  2. opeWHO  3. 13 languages: Arabic, Chinese, English , French, Hindi, Indian sign language, Macedonian, Persian, Portuguese, Russian, Serbian, Spanish, and Turkish  4. no | Introduction to emerging respiratory viruses including COVID-19: methods for detection, prevention, response, and control  - introduction  - detecting COVID  - risk communication and community engagement  - preventing and responding COVID  Level 2 learning objectives | Ecuador (n=9521)  Mexico (n=8236)  Columbia (n=6137)  Chile (n=4067)  Argentina (3609)  India (n=1117)  Peru (n=925)  African region: 7643 (3%) | Number of learners from no-English speaking countries significantly increased once multiple languages were added | No assessment due to rapped dissemination need |
| Margolis et al., 2015 | 1. Latin America  2. na  3. Spanish & Portuguese  4. certificate of participation and certificate of compleation | Immunopathology in native and transplanted kidneys | Mexico 62  Brazil 59  Uruguay 53  Argentina 48  Peru 40  Ecuador 32  Chile 31  Bolivia 27  Costa Rica 24  Cuba 23  Guatemala 23  Venezuela 23  Columbia 13  Dom rep 12  El Salvador 8  Panama 2 | **Barrier**: lack of time to devote to learning. 12 and 6% had difficulties with internet access and technology respectively. | significant knowledge gain between pre-and post-course assessment.88.75% completion rate (measured by course certificate). 86% reported being very satisfied |
| Medina-Presentado et al., 2017 | 1. Latin America  2. na  3. Spanish & Portuguese  4. certificate of participation | Current threats of the main antimicrobial resistances: local and global epidemiological aspects. Resistance mechanisms. Diagnostic approaches in the laboratory: detection methods and interpretation of results. Rational use of antimicrobial agents. Prevention and surveillance measures. •solation and hygiene measures. Outbreak control. Clinical management and new treatments. | Brazil: 264  Argentina: 238  Mexico: 207  Paraguay: 24  Colombia: 23  Venezuela: 22  Ecuador: 21  Peru: 15  Costa Rica: 11  Dominican Republic:7  Salvador: 5  Bolivia: 3  Honduras; 3  Nicaragua: 2  Cuba: 1  Guatemala: 1 | **Facilitators**: course promotion through academic networks | Significant knowledge acquisition assessed in post-course assessment, 46% achieved certification of completion, 54% achieved certificate of participation. High course satisfaction was indicated in post-course survey. Commitment to change were made and followed through by many responders |
| Li & Li, 2016 | 1. China  2. na  3. na  4. na  5. blended in part of university degree | Neurobilogy | All 98 students are Chinese |  |  |
| Millifan & Littljohn, 2016 | 1. na  2. edX  3. na  4. na | developing and conducting clinical trials | Nigeria 1, Egypt 2, Argentina 1, Botswana 1, India 6, Peru 1, Thailand 1, Russia 1, Egypt 1, Brazil 1, Uganda 1 |  |  |
